# Neuropathic Pain in Neuromyelitis Optica Spectrum Disorders: Prevalence and Management Strategies—A Systematic Review and Meta-Analysis

**DOI:** 10.3390/jcm15041378

**Published:** 2026-02-10

**Authors:** Alexandra Akrivaki, Vasileios Giannopapas, Evangelia-Makrina Dimitriadou, Dimitrios Tzanetakos, Dimitrios Kitsos, Konstantina Stavrogianni, Athanasios K. Chasiotis, Georgios Tsivgoulis, John S. Tzartos, Sotirios Giannopoulos

**Affiliations:** 1 Second Department of Neurology, Attikon University Hospital, National & Kapodistrian University of Athens, Rimini 1, 12462 Athens, Greece; alexandra.akrivaki@gmail.com (A.A.); bgiannopapas@gmail.com (V.G.); evadim93@hotmail.gr (E.-M.D.); tzanetakosdim@yahoo.com (D.T.); stavrogianni.k@gmail.com (K.S.); thanosch1@gmail.com (A.K.C.); tsivgoulisgiorg@yahoo.gr (G.T.); jtzartos@gmail.com (J.S.T.); 2 Department of Physiology, Faculty of Medicine, School of Health Sciences, University of Ioannina, 45110 Ioannina, Greece; 3 Laboratory of Neuromuscular and Cardiovascular Study of Motion-LANECASM, University of West Attica, 12243 Attica, Greece

**Keywords:** neuromyelitis optica spectrum disorder, NMOSD, neuropathic pain, management

## Abstract

**Introduction:** Neuropathic pain (NP) in neuromyelitis optica spectrum disorder (NMOSD) represents a significant and often under-recognized complication arising from central nervous system (CNS) lesions. Unlike other demyelinating disorders, NMOSD involves a distinct immunopathogenesis primarily driven by aquaporin-4 antibodies (AQP4-IgG), leading to severe inflammatory damage. NP is typically the consequence of inflammatory damage to the spinothalamic tract or dorsal columns, resulting in both acute and chronic pain syndromes. **Methods:** A systematic review and meta-analysis were conducted following a comprehensive search of Medline and Scopus, identifying nine eligible studies reporting on NP in NMOSD. **Results:** Pooled prevalence was estimated using a random-effects metaprop meta-analysis with Freeman–Tukey transformation and REML-based heterogeneity estimation. The pooled prevalence of NP among patients with NMOSD was 56.2% (95% CI: 41.7–70.1%; I^2^ = 95.3%, *p* < 0.001). Sensitivity analysis including only AQP4-IgG^+^ cohorts revealed a prevalence of 63.2% (95% CI: 23.4–94.7%; I^2^ = 98.1%, *p* < 0.001). No significant difference was found between mixed and AQP4-IgG^+^-only populations (53.05% vs. 63.27%, *p* = 0.63). Meta-regression showed no significant associations between NP prevalence and age (β = 0.01, *p* = 0.33) or disability (β = 0.08, *p* = 0.18). Qualitative synthesis demonstrated an association between thoracic spinal cord lesions and NP, and also indicated that NP was often resistant and refractory to standard pharmacologic therapies. **Conclusions:** NP affects one in two NMOSD patients, and is associated with thoracic spinal cord lesions. In comparison with multiple sclerosis, NP in NMOSD is primarily structural and immunopathological in origin. Treatment strategies remain inadequate, emphasizing the need for early recognition and a disease-specific therapeutic approach.

## 1. Introduction

Neuromyelitis optica spectrum disorder (NMOSD) is a rare, severe, immune-mediated disorder of the central nervous system (CNS), characterized by recurrent attacks of optic neuritis, longitudinally extensive transverse myelitis, and area postrema syndrome [1]. In contrast to multiple sclerosis (MS), NMOSD pathophysiology includes autoantibodies directed against aquaporin-4 (AQP4-IgG), targeting predominantly astrocytes. However, a subset of patients may be seronegative [1].

Neuropathic pain (NP) has been identified as a common and debilitating symptom of NMOSD which contributes to reduced quality of life [2]. Pain is typically a result of extensive inflammatory lesions involving the spinal cord, particularly the spinothalamic tracts and dorsal columns, although brainstem structures may also be affected [3]. NP may manifest as persistent burning sensations, allodynia, and dysesthesia, often presenting acutely during inflammatory attacks or emerging later in the disease course [3].

While the underlying pathophysiological mechanisms may not be totally understood, NP in NMOSD is believed to stem from astrocyte damage, secondary demyelination, axonal injury, and maladaptive central sensitization processes [3]. In addition to accumulating disability due to relapses, NMOSD patients experience further deterioration in domains such as quality of life (QoL) and activities of daily life, due to chronic neuropathic pain [2]. Importantly, pain symptoms in NMOSD are frequently refractory to standard analgesic treatments, dictating the need for research in targeted immunotherapies and adjunctive pain management strategies [3].

Despite its recognized clinical burden, reported estimates of the prevalence of neuropathic pain in NMOSD vary across published studies, likely reflecting differences in study design, patient populations, and assessment approaches. A systematic synthesis of the available evidence is therefore required, to better define the overall prevalence of neuropathic pain in NMOSD. Accordingly, the aim of this systematic review and meta-analysis is to consolidate current evidence, provide a precise estimate of NP prevalence in NMOSD, describe its clinical phenotypes, and discuss challenges in management.

## 2. Methods

### 2.1. Protocol Registration

This systematic review and meta-analysis was prospectively registered with the Open Science Framework (OSF.IO/95EHV). Due to the nature of this study, neither ethics board approval nor informed patient consent were required.

### 2.2. Literature Search Strategy

The systematic review and meta-analysis were conducted in accordance with the Preferred Reporting Items for Systematic Reviews and Meta-Analysis (PRISMA) guidelines [4], and written according to the MOOSE guideline [5]. The completed PRISMA 2020 checklist is provided in Supplementary Figure S3. A comprehensive systematic literature search of the MEDLINE PubMed and Scopus databases was performed by two independent authors (AA, VG). Additionally, the first 200 search results from Google Scholar were included in the literature search to identify additional relevant studies and grey literature. Search queries included “neuromyelitis Optica spectrum disorders”, “NMOSD”, “Devic”, and “neuropathic pain”. The complete search algorithm is provided in the Supplementary Materials. The search spanned from inception until 20 April 2025, with no language or year-of-publication restrictions. Retrieved records were exported, duplicates were removed, and studies were screened in two stages (title/abstract screening followed by full-text assessment). Randomized controlled trials, observational studies and case-control studies were considered eligible for consideration.

### 2.3. Inclusion and Exclusion Criteria

On the basis of the pre-defined criteria, studies were included if they (a) comprised patients with a definite NMOSD diagnosis, (b) comprised adult participants (age > 18 years), and (c) reported a number of NP cases, while studies were excluded if they (a) involved no definite diagnosis of NMOSD, (b) used purposive sampling, (c) did not include and/or report the number of participants that were AQP4-IgG^+^, and (d) did not report the NP prevalence. Study selection and consequent data extraction was performed by two independent authors (AA, VG), with potential disagreements being resolved by the corresponding author (SG).

### 2.4. Outcomes

The primary outcome of this study was the prevalence of NP among patients with NMOSD. A pooled prevalence estimate was calculated using meta-analytical techniques when at least four studies reported NP prevalence data in NMOSD populations. Secondary outcomes included the assessment of potential associations between NP prevalence and age, disability status, and disease duration, where sufficient data were available. Additional outcomes comprised a qualitative synthesis of reported pharmacological interventions and proposed underlying mechanisms of neuropathic pain in NMOSD.

### 2.5. Quality Assessment

Bias assessment of potential eligible studies was conducted using the Risk Of Bias of Non-Randomized Studies (Robins-I) [6] by two independent authors (AA, VG). Potential disagreements were resolved by the senior author (SG).

### 2.6. Statistical Analysis

An aggregated meta-analysis of the pooled prevalence of NP in patients with NMOSD was performed using R-studio 2025.09.2 for IOS. The pooled prevalence of NP in NMOSD patient was produced by the random effects model of the “meta-prop” function of R-Meta package using the Freeman–Tukey double arcsine transformation. The between-study variance (τ^2^) in the random-effects model was estimated using the restricted maximum likelihood (REML) method, as it provides statistically efficient and robust estimation of heterogeneity, particularly in the presence of between-study variability. Results are presented in the following format: %, 95% CI [%. %] [7]. Heterogeneity between included studies was assessed with Cochran Q (sig. level 0.1) and I^2^ statistics. Publication bias across individual studies was assessed in cases where more than four studies were included in each analysis, using funnel plot inspection and Egger’s linear regression test [8,9]. Potential associations between the main outcome (pooled prevalence) and age, disability status and disease duration were assessed by meta-regression (“meta-reg” function, r-meta) in cases where more than five studies reported relevant data [10,11].

## 3. Results

### 3.1. Literature Search and Included Studies

**Figure 1:**
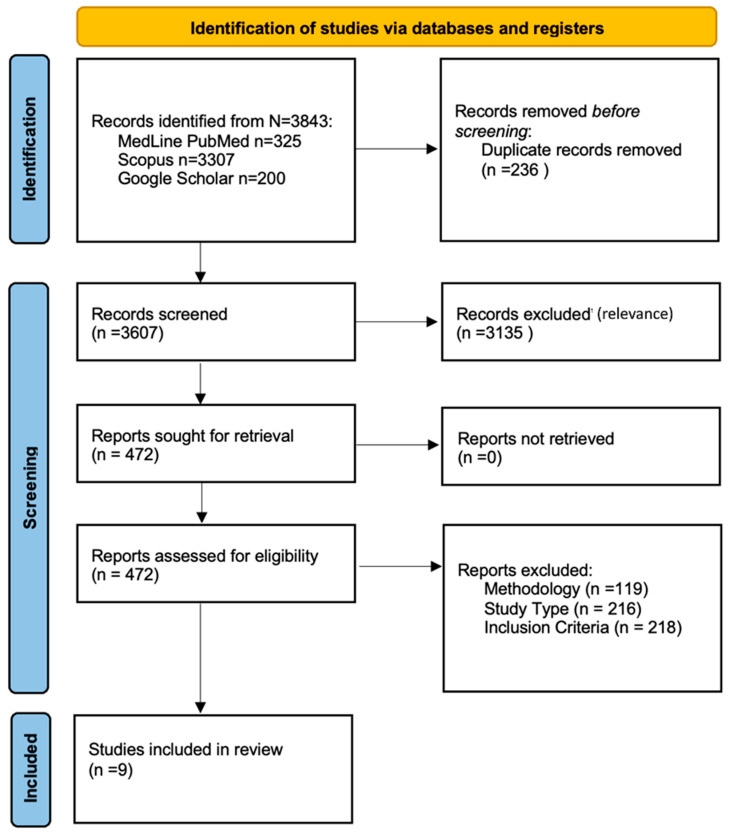
PRISMA flowchart.

The systematic literature search yielded a total of 3843 records. After the removal of duplicate records, and the implementation of inclusion-exclusion criteria, nine studies were deemed eligible for consideration (Figure 1).

### 3.2. Results of Quality Assessment

Eligible studies underwent quality assessment using the ROBINS-I tool (Supplementary Figure S1). The overall risk of bias was found to be low, although some studies presented potential bias in outcome measurement.

### 3.3. Quantitative Analysis

#### 3.3.1. Primary Outcomes

**Figure 2:**
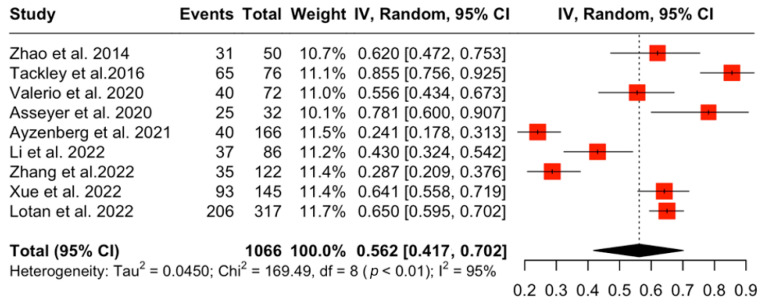
Prevalence of NP in NMOSD patients [12,13,14,15,16,17,18,19,20].

**Figure 3:**
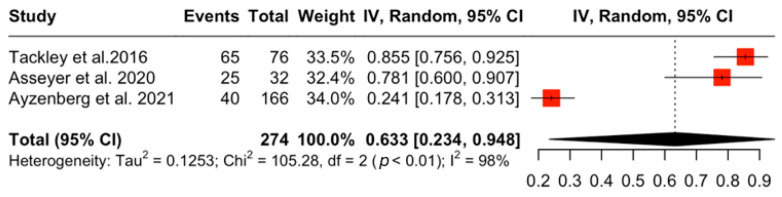
Prevalence of NP in AQP4-IgG^+^ NMOSD patients [12,15,16].

**Figure 4:**
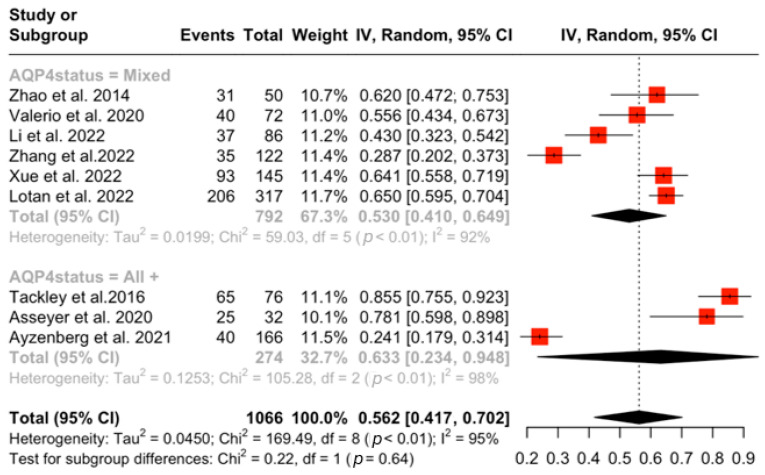
Subgroup differences in NP prevalence between AQP4-IgG^+^ and mixed AQP status NMOSD patients [12,13,14,15,16,17,18,19,20]. Grey text indicates subgroup headings and subgroup-specific pooled estimates with associated heterogeneity measures.

A total of nine studies [12,13] with NMOSD patients were included (Table 1). The aggregated pooled prevalence of NP in NMOSD patients was 56.2% (95% CI: [41.7%, 70.1%], I^2^ = 95.3, *p* < 0.001) (Figure 2). A subsequent sensitivity analysis of prevalence was performed including only studies that had AQP4-IgG^+^ participants. The aggregated pooled prevalence of NP in AQP4-IgG^+^ NMOSD patients was 63.2% (95%CI: [23.4%, 94.7%], I^2^ = 98.1%, *p* < 0.001) (Figure 3). Finally, a subgroup analysis was performed comparing studies that included mixed serostatus (AQP4-IgG^+^ and AQP4-IgG^−^) and studies that included only AQP4-IgG^+^ NMOSD patients; this showed no statistically significant difference between the two groups (53,05% vs. 63.27%, *p* = 0.63) (Figure 4). Corresponding results using the DerSimonian–Laird (DL) estimator are shown in the Supplementary Materials (Supplementary Figures S2–S4) [14].

**Table 1:** Characteristics of included studies.

Author and Year	Sample Size	AQP4-IgG^+^ %	Cases of NP	Mean Age	Mean EDSS	Mean Disease Duration	NP Assessment
Tackley et al. 2016 [12]	76	100%	65	54.6	-	10.4	PSI
Xue et al. 2022 [21]	145	73.1%	93	44.9	3.5	-	-
Li et al. 2022 [19]	86	85%	37	46.2	4.3	-	-
Ayzenberg et al. 2021 [15]	166	100%	40	51.7	3.5	9.1	PDQ
Asseyer et al. 2020 [16]	32	100%	25	51.1	4	6.3	PDQ
Valerio et al. 2020 [18]	72	33%	40	43.3	5.5	-	BPI, NPSI, DN4, MPQ
Zhao et al. 2014 [17]	50	82%	31	49	4.7	-	-
Zhang et al. 2022 [20]	122	96%	35	43.3	1.6	5.19	BPI, DN4, NPSI
Lotan et al. 2022 [13]	317	67%	206	43.9	-	-	-

BPI—Brief Pain Inventory, DN4—Douleur Neuropathique 4, EDSS—Expanded Disability Severity Scale, MPQ—Short-form McGill Pain Questionnaire, NP—Neuropathic Pain, NPSI—Neuropathic Pain Symptom Inventory, PDQ—Pain Detect Questionnaire, PSI—Pain Severity Index.

#### 3.3.2. Secondary Outcomes

Meta-regression techniques were employed to assess potential associations between the prevalence of NP and participants’ age and disability status. There were no statistically significant associations between NP prevalence and age (β = 0.01, *p* = 0.33) or Expanded Disability Status Scale (EDSS) scores (β = 0.08, *p* = 0.18).

#### 3.3.3. Publication Bias

Based on funnel plot inspection (Supplementary Figure S6) and the results of the Egger’s linear regression test (β = 1.64, *p* = 0.76), there was evidence of a moderate degree of publication bias.

### 3.4. Qualitative Analysis

#### 3.4.1. Lesion Topography and Its Impact on NP in NMOSD Patients

Lesion localization within the spinal cord seems to play a crucial role in the development and severity of NP in NMOSD. Several studies reported a strong association between thoracic spinal cord involvement and NP occurrence [12,15,19,21]. In particular, Tackley et al. (2016) reported that patients with thoracic lesions were more likely to experience severe, chronic pain than those with cervical lesions [12]. Additionally, thoracic lesions extending to four or more vertebral segments were independently associated with a markedly increased risk of NP [19]. Upper thoracic lesions were specifically implicated to play a role in the occurrence and intensity of pain symptoms, while cervical and lower thoracic lesions were not as strongly associated [15]. Apart from the abovementioned spinal involvement, potential alterations in central pain processing have also been explored, although evidence remains limited. Asseyer et al. (2022) identified an inverse correlation between the volume of the ventral posterolateral thalamic nucleus (VPN) and pain intensity, suggesting a central modulation component [16]. However, not all studies consistently support these associations. Valerio et al. (2020) did not report any significant association between lesion location and pain severity [18]. Additionally, Zhao et al. (2014), reported no significant difference in mean lesion length between patients with and without NP [17].

#### 3.4.2. Clinical Presentation of NP in NMOSD Patients

The most reported distribution of NP in NMOSD involved the lower limbs and trunk [12,21], sensory symptoms including continuous superficial burning pain, paresthesia and dysesthesia [18], while pain onset varied across patients. In Xue et al.’s cohort (2022), NP was the initial symptom in more than half of NMOSD patients, while in others it appeared later in the disease course [21]. The duration of NP varied significantly, ranging from two weeks to over 16 years as reported by Xue et al. (2022) [21]. Moreover, Tackley et al. (2016) reporting a mean duration of 6 years, underscoring the chronic nature of the condition [12,21]. Regarding relevant comorbidities, Zhang et al. (2022) identified an association between NP and depression, highlighting a potential psychosomatic interplay [20]; however, this finding was not supported by other studies [16].

#### 3.4.3. Management and Treatment of NP

Management of NP in NMOSD presented significant heterogeneity, with notable variability in prescribing practices, treatment uptake, and reported effectiveness across studies. Xue et al. (2022) reported that 65.6% of patients received medications broadly used for NP in other conditions, these medications including gabapentin, pregabalin, carbamazepine, duloxetine, and tricyclic antidepressants, yet the response rate was only 46% [21]. Similarly, Lotan et al. (2023) observed that the most prescribed medications were gabapentin, baclofen, pregabalin, acetaminophen and duloxetine [13]. Among symptomatic treatments, medical cannabis was rated as the most effective, with 63% of patients describing it as ‘very helpful’, whereas acetaminophen was rated the least helpful [13]. Despite widespread medication use, Li et al. (2022) highlighted that a significant proportion of patients with NP received limited analgesic intervention, and even among treated individuals responses varied [19]. Immunotherapy also appears to modulate NP severity. Ayzenberg et al. observed a 40% retrospective reduction in pain intensity among patients treated with rituximab, azathioprine, mycophenolate mofetil, or tocilizumab, suggesting that targeted immunosuppression may alleviate pain by reducing ongoing inflammatory activity [15]. Xue et al. similarly reported that 9/21 patients with refractory NP improved following rituximab, and 3/11 following tocilizumab, although transient pain worsening was noted following IL-6 blockade in some cases [21].

This finding may suggest that a potentially effective treatment mechanism targeted the underlying disease pathology, thereby reducing chronic pain burden more effectively than symptomatic treatments alone [15]. Collectively, these findings highlight the need for structured, stepwise treatment pathways, as medication responses vary and many patients cycle through multiple symptomatic therapies before achieving partial relief.

#### 3.4.4. Comparison of NP Between NMOSD and MS

NP is common in both NMOSD and MS, yet important distinctions exist in its pathogenesis and clinical presentation. To begin with, Tackley et al. (2016) proposed that NMOSD patients may experience more severe pain due to predominant thoracic spinal cord involvement; in MS, in contrast, cervical lesions are more frequent [12]. Damage to thoracolumbar autonomic nuclei in NMOSD could dysregulate ascending pain pathways, enhancing the qualitative experience of pain. In addition, molecular mechanisms also differ. Li et al. (2022) highlighted that AQP4 loss in NMOSD disrupts glutamate uptake and GABAergic neurotransmission, thus promoting excitotoxicity and development of chronic pain [19]. Reactive astrocyte phenotypes may further modulate this neuroinflammatory environment, intensifying pain susceptibility (notably, A1 astrocytes result in excitotoxicity, while A2 astrocytes may have a neuroprotective function) [19]. Contrarily, psychological comorbidities appear less pronounced in NMOSD. Asseyer et al. (2022) found no significant association between NP and depression in NMOSD [16], in contrast to MS where pain frequently correlates with mood disorders [22]. This suggests NP in NMOSD may be more directly linked to structural injury rather than psychological amplification. Collectively, these findings emphasize that NP in NMOSD is more anatomically and immunologically driven, whereas in MS both structural and psychological factors contribute significantly to pain expression.

#### 3.4.5. Painful Tonic Spasms

Although the present meta-analysis focused on neuropathic pain, painful tonic spasms (PTSs) represent an important and highly prevalent pain manifestation in NMOSD. Among the nine studies included in the systematic review, three reported data on PTS in addition to neuropathic pain. In these studies, PTSs were described as recurrent, brief, and often severely painful muscle contractions, typically associated with spinal cord involvement. In the study by Ayzenberg et al., PTSs were reported in approximately one quarter of patients with chronic NMOSD-associated pain (26.4%) [15]. Valerio et al. identified PTSs across different pain subgroups, including in patients classified as having neuropathic pain, non-neuropathic pain, and no pain, suggesting that PTSs may occur independently of neuropathic pain classification [18]. In the cohort described by Xue et al., PTSs were observed in 21 patients (17.9%) and were consistently associated with myelitis-related spinal cord involvement, most commonly occurring during the convalescent stage following acute myelitis [21]. In this cohort, the majority of patients with PTS received antiepileptic medications, including carbamazepine, oxcarbazepine, or phenytoin, with a high reported rate of symptomatic response of 85% [21].

## 4. Discussion

NP is a highly prevalent disabling symptom in patients with NMOSD. In this meta-analysis, the pooled prevalence of NP in NMOSD patients was 56.2% with an even higher prevalence of 63.2% observed in AQP4-IgG seropositive cohorts. However, subgroup analysis revealed no statistically significant difference in NP prevalence between mixed and exclusively AQP4-IgG-positive populations. These findings emphasize that NP, regardless of serostatus, is a core component of the symptomatology of NMOSD.

Meta-regression analyses indicated that NP prevalence was not significantly associated with either age or disability status, as measured by EDSS. This suggests that NP may be present independently of global neurological disability and functional status, implying that specific lesion localization and neurobiological mechanisms play a more decisive role in its development. Qualitative synthesis further analyzes these implications. Spinal cord involvement, particularly when affecting the thoracic segments, emerged as a critical determinant of NP. Several studies consistently demonstrated that thoracic lesions, especially when extensive (≥4 vertebral segments), were associated with a markedly higher risk and severity of NP [12,15,19,21]. These findings suggest a predilection of NMOSD-related inflammatory damage for disrupting key nociceptive and autonomic pathways within the thoracolumbar cord (e.g., ascending lamina I neurons of the spinal dorsal horn).

Management strategies were heterogeneous and often suboptimal. Anticonvulsants such as gabapentin and pregabalin were the most commonly prescribed agents, along with tricyclic antidepressants and duloxetine [13,21]. However, therapeutic response rates were modest, with several studies reporting effective pain control in less than half of treated patients [13,21]. Immunotherapy targeting inflammatory activity, such as rituximab, may attribute to indirect benefits on pain symptoms, but direct evidence remains limited [15].

**Figure 5:**
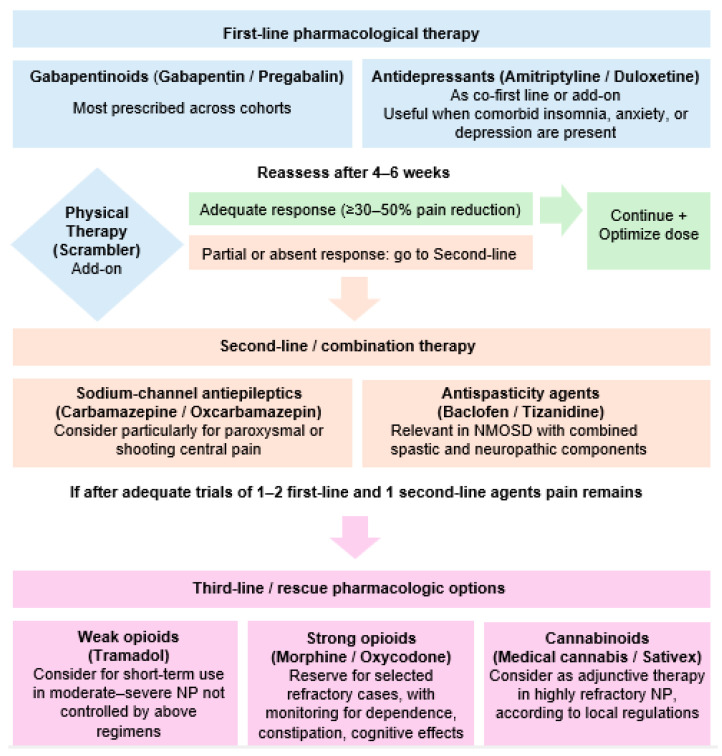
Proposed therapeutic algorithm for the management of NP in NMOSD, based on the most frequently reported agents.

Based on the aggregated quantitative and qualitative evidence from the included studies, we developed a conceptual treatment algorithm (Figure 5) outlining first-line, second-line, and rescue pharmacologic options. This framework reflects the real-world patterns observed across cohorts, and emphasizes the importance of individualized, multimodal pain management in NMOSD. Beyond pharmacological interventions, a recent single-blind, sham-controlled trial by Mealy and colleagues 2020, reported improvement in neuropathic pain severity and also in depression symptomatology after 10 consecutive scrambler therapy sessions targeting dermatomes adjacent to the region of NP [23], highlighting the potential benefit of non-pharmacological, physical therapy interventions in the management of NP in NMOSD.

The persistent undertreatment of NP, as highlighted across multiple studies, represents an urgent unmet clinical need that can potentially have a significant effect on patients’ quality of life. Regarding non-pharmacological interventions for the management of NP of CNS origin, neuromodulation techniques such as (electro) acupuncture and transcutaneous electric nerve stimulation have demonstrated a significant effect in alleviating central NP in patients with spinal cord injury and MS [23,24]. In parallel, psychological interventions, particularly cognitive behavioral therapy, have been shown to improve sleep quality and mood, and reduce pain-catastrophizing, in individuals with NP [25,26].

When comparing NP between NMOSD and MS, distinct differences emerge. In the case of MS, both structural and psychological factors contribute significantly to pain expression; in contrast, NMOSD-associated NP appears more anatomically and immunologically driven [12,16,22]. Thoracic spinal cord lesions predominate in NMOSD and disrupt autonomic and nociceptive pathways at a critical level, whereas cervical cord involvement is more typical in MS [12]. On a molecular level, the loss of AQP4 in NMOSD alters glutamate and GABA neurotransmission, creating a pro-excitatory environment that fosters chronic pain [19]. These differences have important therapeutic implications and highlight the necessity for disease-specific pain management strategies.

Although this meta-analysis focused on neuropathic pain, converging evidence indicates that PTS represent a frequent, clinically distinct, and highly disabling pain manifestation in NMOSD that is closely linked to spinal cord involvement [15,21,27,28,29]. Across independent cohorts, PTS are reported in approximately 22–44% of patients with NMOSD, occurring almost exclusively in association with myelitis, particularly longitudinally extensive transverse myelitis, and often emerging during the convalescent phase following acute spinal cord inflammation [15,21,27,28,29]. These studies consistently demonstrate that PTS exert a profound negative impact on daily functioning and quality of life, with the vast majority of affected patients requiring symptomatic treatment [27,28,29]. From a therapeutic perspective, there is broad agreement that sodium-channel-blocking antiepileptic drugs, particularly carbamazepine and oxcarbazepine, are highly effective for symptomatic control, whereas gabapentinoids show comparatively lower efficacy [21,27,28]. Collectively, these findings support the notion that PTS constitute a pain-related manifestation that is pathophysiologically and clinically distinct from neuropathic pain, reflecting corticospinal tract dysfunction and spasticity rather than primary somatosensory system damage. Painful tonic spasms should therefore be considered an important area for future research, particularly with respect to standardized definitions and assessment frameworks.

The present study has some limitations. First, there was significant heterogeneity observed across studies, likely reflecting differences in diagnostic criteria for NP, imaging protocols, and assessment tools. Second, the cross-sectional design of most included studies limits causal inferences regarding lesion localization and pain development, while interpretations of anatomic topographic brainstem correlates of pain intensity are constrained by the very limited available literature, which is currently based on a single report. Third, although this meta-analysis provides pooled prevalence estimates, detailed analyses on pain severity grading and longitudinal outcomes were limited by available data. Furthermore, a moderate degree of publication bias was identified. Nonetheless, the consistency of findings across studies strengthens the robustness of these results. Finally, an important limitation that needs to be acknowledged is the evolving classification of CNS autoimmune disorders: the proposed diagnostic criteria for Myelin oligodendrocyte glycoprotein (MOG) antibody-associated disease were only established in 2023 [30]. Prior to this, individuals with MOG-IgG-associated optic neuritis or myelitis may have been classified under the NMOSD spectrum, potentially introducing diagnostic heterogeneity across included studies. This is reflected in the results of the subgroup meta-analysis between studies that included mixed AQP4-IgG serostatus and studies that included only AQP4-IgG^+^ NMOSD patients.

## 5. Conclusions

This is the first systematic review and meta-analysis to examine the prevalence of NP in NMOSD patients. On the basis of our results, it may be said that NP is a highly prevalent, persistent, and burdensome condition in NMOSD, affecting more than one in two NMOSD patients, and that it is closely associated with thoracic spinal cord involvement and AQP4-IgG-mediated immunopathology. Management remains challenging, with current treatments offering only partial relief. Distinct pathophysiological mechanisms distinguish NP in NMOSD from that in MS, underscoring the need for tailored diagnostic and therapeutic approaches. Future longitudinal studies focusing on early identification and targeted intervention for NP in NMOSD are warranted to improve patient outcomes.

## Data Availability

The datasets generated and/or analyzed during the current study are available from the corresponding author upon reasonable request.

## Supplementary Materials

The following supporting information can be downloaded at: https://www.mdpi.com/article/10.3390/jcm15041378/s1. Figure S1: ROBINS-I traffic plot. Figure S2: Prevalence of NP in NMOSD patients (DL estimation). Figure S3: PRISMA Checklist. Figure S4: Prevalence of NP in AQP4-IgG⁺ NMOSD patients (DL estimation). Figure S5: Subgroup difference in NP prevalence between AQP4-IgG⁺ and mixed AQP status NMOSD patients (DL estimation). Figure S6: Funnel plot.

## Abbreviations

The following abbreviations are used in this manuscript:

AQP4-IgG	Aquaporin-4 antibodies
CNS	Central nervous system
EDSS	Expanded disability status scale
MOG	Myelin oligodendrocyte glycoprotein
MS	Multiple sclerosis
NMOSD	Neuromyelitis optica spectrum disorder
NP	Neuropathic pain
PTS	Painful tonic spasms
PRISMA	Preferred reporting items for systematic reviews and meta-analysis
QoL	Quality of life
